# Lesion Stiffness Measured by Magnetic Resonance Elastography: A Novel Biomarker for Differentiating Benign, Premalignant and Malignant Prostate Lesions

**DOI:** 10.3390/diagnostics15202603

**Published:** 2025-10-16

**Authors:** Süheyl Poçan, Levent Karakaş

**Affiliations:** 1 BHT Clinic Istanbul Tema Hospital, Atakent Mahallesi, 4. Cadde, No: 36, Küçükçekmece, 34307 Istanbul, Türkiye; 2 Department of Radiology, Faculty of Medicine, Nişantaşı University, Maslak Mahallesi, Taşyoncası Sokak, No: 1V ve No: 1Y, Sarıyer, 34398 Istanbul, Türkiye; 3 Department of Radiology, Gaziosmanpaşa Training and Research Hospital, 34255 Istanbul, Türkiye; drleventkarakas@hotmail.com

**Keywords:** lesion stiffness, magnetic resonance elastography, prostate cancer, prostate imaging reporting and data system

## Abstract

**Background/Objectives**: This study aimed to assess whether magnetic resonance elastography (MRE)-derived stiffness measurements of the central gland, entire gland, and lesions of the prostate differ among benign, premalignant, and malignant lesions and to evaluate their diagnostic performance in distinguishing these groups. **Methods**: This prospective study enrolled 113 men (mean age, 62.7 ± 7.2 years). Patients were categorized into benign (*n* = 75), premalignant (*n* = 15; atypical small acinar proliferation and high-grade prostatic intraepithelial neoplasia), and malignant (*n* = 23; adenocarcinoma) lesion groups based on histopathological findings. MRE-derived stiffness was measured at the lesion, central gland, and entire gland levels. Other evaluated parameters included diffusion restriction, contrast retention, prostate-specific antigen (PSA) levels, prostate volume, and Prostate Imaging Reporting and Data System (PI-RADS) score. **Results**: Mean central gland stiffness did not differ between benign and premalignant lesions, but was markedly higher in the malignant group (Benign: 3.3 ± 0.2 vs. Premalignant: 3.4 ± 0.2 vs. Malignant: 3.6 ± 0.3 kPa; *p* < 0.001). A similar pattern was observed for entire gland stiffness (Benign: 3.3 ± 0.4 vs. Premalignant: 3.3 ± 0.4 vs. Malignant: 4.1 ± 0.6 kPa; *p* < 0.001). Median lesion stiffness increased stepwise from benign to premalignant to malignant lesions (Benign: 3.6 vs. Premalignant: 5.8 vs. Malignant: 7.7 kPa; *p* < 0.001). Central and entire gland stiffness distinguished malignant lesions but failed to differentiate premalignant lesions from benign lesions. Lesion stiffness demonstrated superior diagnostic accuracy in distinguishing premalignant from benign (AUC 0.82; accuracy 83.3%) and malignant lesions from premalignant lesions (AUC 0.86; accuracy 82.5%) compared to central and entire gland stiffness. **Conclusions**: MRE-derived lesion stiffness is a promising diagnostic biomarker, effectively distinguishing benign, premalignant, and malignant prostate lesions. Prostate gland stiffness measured by MRE, especially lesion-specific measurements, may be considered as an additional candidate procedure that can be accommodated in multiparametric magnetic resonance imaging.

## 1. Introduction

Prostate cancer (PCa) is the most common cancer and the third leading cause of cancer-related deaths among men worldwide [1]. In 2020 alone, over 1.4 million new PCa cases were recorded globally, and approximately 375,000 men died from the disease [2]. Although early diagnosis and treatment dramatically reduce mortality [3], conventional diagnostic methods, such as prostate-specific antigen (PSA) measurements, digital rectal examination, and assessment of lower urinary tract symptoms, lack specificity due to overlap with benign disorders, including prostatitis and benign prostatic hyperplasia (BPH) [4]. Thus, PSA-based screening can lead to unnecessary biopsies and overdiagnosis of indolent disease [5]. Confirmatory diagnosis of PCa relies on histopathological evaluation of prostate tissue obtained via biopsy. Most PCas are acinar adenocarcinomas, and grading by the Gleason score (now reported as International Society of Urological Pathology Grade Groups 1–5) is central for assessing tumor aggressiveness [6]. Notably, high-grade prostatic intraepithelial neoplasia (HGPIN) is a common premalignant lesion of the prostate and is considered a precursor to invasive carcinoma [7]. However, prostate biopsies are associated with a significant risk of complications and incorrect sampling [8]. These limitations highlight the urgent need for more specific PCa markers, prompting increased interest in advanced imaging techniques for their identification.

In recent years, multiparametric magnetic resonance imaging (mpMRI) has emerged as a cornerstone in PCa diagnosis and risk stratification. mpMRI, which is used in the noninvasive evaluation of the prostate, has provided advanced opportunities for tumor detection, staging, localization, characterization, risk classification, surveillance, and image-guided biopsy [9,10]. Standardized mpMRI protocols, as outlined by the Prostate Imaging and Reporting Data System (PI-RADS) guidelines, aim to improve diagnostic accuracy and consistency across imaging centers [11]. Furthermore, mpMRI includes T2-weighted imaging to assess tumor morphology, diffusion-weighted imaging to assess cell density, and dynamic contrast-enhanced imaging to assess vascularization [12]. Studies assessing the diagnostic performance of mpMRI for detecting PCa have shown sensitivities and specificities ranging from 70% to 90% [13,14,15]. This wide range suggests that mpMRI findings can overlap among benign, premalignant, and malignant lesions and may expose a substantial proportion of patients to unnecessary biopsies. This, particularly in the presence of anatomical variations and other benign pathologies, raises concerns regarding the diagnostic accuracy of mpMRI in detecting PCa [16,17]. Thus, while mpMRI has advanced PCa diagnostics, there remains an unmet need for adjunct imaging techniques to further improve lesion characterization and differentiate cancer from non-cancerous abnormalities.

Magnetic resonance elastography (MRE) has recently been introduced as a novel MRI-based technique for the quantitative assessment of tissue stiffness in vivo [18]. MRE is a phase-contrast MRI method that visualizes mechanically induced shear waves in tissues and reconstructs quantitative elastograms reflecting the shear modulus (stiffness) of the tissue [19]. In essence, an external driver generates low-frequency vibrations, and MRI captures the resulting tissue displacements; stiffer regions deform less and thus exhibit higher apparent shear-wave speeds or moduli. Because many tumors induce a desmoplastic reaction or have intrinsically higher cell density and extracellular matrix content, cancerous tissue tends to be mechanically harder than normal or benign tissues. This principle has been validated in several organs, including hepatic fibrosis staging using MRE, and has been studied in the breast, brain, lung, and other tissues [20,21,22]. Thus, tissue stiffness measured by MRE holds promise as a biomarker for distinguishing malignant from benign prostate lesions.

Large-scale clinical validation is required before incorporating MRE-based stiffness measurements into standard mpMRI protocols. While preliminary studies suggest MRE’s potential utility in detecting and characterizing PCa [23,24,25], the number of studies investigating this relationship remains limited. Initial prostate MRE studies have indicated that pathological changes correlate with increased tissue stiffness. Normal prostate tissue typically exhibits baseline stiffness values of a few kilopascals [26,27]. In contrast, whole prostate gland stiffness is reported to range between 4.4 and 4.7 kPa in patients with BPH lesions but between 6 and 8 kPa in patients with malignant lesions [19,28,29]. These studies have focused on technical feasibility, whole-gland stiffness, and discrimination between benign and malignant lesions. However, to the best of our knowledge, no study has focused on comprehensive MRE measurements, including lesion stiffness, for differentiating malignant lesions from premalignant lesions.

High lesion stiffness correlates with tumor aggressiveness in multiple malignancies, including PCa [30]. By directly reflecting the biomechanical properties of a lesion, MRE may help identify which MRI-suspicious areas are likely to be malignant and which are more consistent with benign conditions or indolent changes. We hypothesized that quantitative stiffness, particularly lesion stiffness, measured by MRE, can help distinguish benign lesions and premalignant lesions from malignant lesions in the prostate. Therefore, this study aimed to investigate whether MRE-derived stiffness measurements of the central gland, entire gland, and lesions of the prostate differ among benign, premalignant, and malignant lesions and to evaluate their diagnostic performance in distinguishing these groups.

## 2. Materials and Methods

This prospective study included patients who underwent mpMRI and MRE for suspected PCa at the Urology Department of BHT Clinic Istanbul Tema Hospital between March and November 2023. All procedures adhered to the ethical standards outlined by the Institutional Research Committee and were aligned with the 1964 Helsinki Declaration and its subsequent amendments. This study was approved by the Ethics Committee of Nişantaşı University (15 March 2023, No: 2023/11). Written informed consent was obtained from all the participants.

### 2.1. Study Population

**Figure 1:**
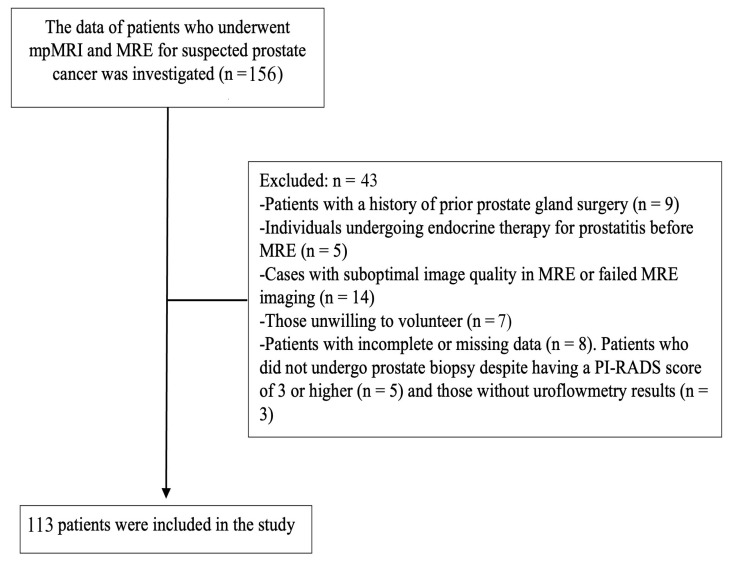
Flowchart of the study.

During the study period, 156 patients were assessed for study eligibility. The inclusion criteria were patients exhibiting lower urinary tract symptoms, such as a diminished urinary stream or difficulties in urination, coupled with elevated PSA levels. Additionally, patients with normal or high total or free PSA levels, those showing stiffness upon rectal examination, and those referred from other departments owing to elevated total or free PSA levels were considered for inclusion. Another prerequisite was that the patient had undergone mpMRI and MRE examinations for suspected PCa. The exclusion criteria were a history of prostate gland surgery, individuals undergoing endocrine therapy for prostatitis before MRE, cases with suboptimal image quality in MRE or failed MRE imaging, those unwilling to volunteer, and patients with incomplete or missing data. Causes of suboptimal image quality: Breath and motion artifacts, as well as artifacts caused by residual stool in the distal colon and rectum, despite colon cleansing prior to the procedure. Patients were required to hold their breath during the MRE. Patients who could not tolerate the pressure exerted by the passive driver of elastography and were unable to hold their breath appropriately were excluded from the study. The study flowchart is presented in Figure 1.

### 2.2. Study Protocol

The ages of the participants at baseline were recorded. All participants underwent a standard diagnostic protocol for suspected PCa, including PSA measurement, mpMRI, and prostate biopsy when indicated.

### 2.3. Laboratory Measurements

The total blood PSA values quantified at the time of initial admission were included in the study. The certified biochemistry laboratory at our hospital performed the measurements using routinely calibrated devices. The average flow rate, maximum flow rate, and volume of all participants were measured using a conventional uroflowmetry device (Oruflow, Oruba Technology&Innovation, Ankara, Turkey).

### 2.4. Radiological Measurements

Before biopsy, all patients underwent conventional mpMRI and MRE using a 3.0 T MRI scanner (General Electric Signa Architect; General Electric Healthcare, Waukesha, WI, USA). Patients were positioned supine, head first, with a 40-channel body surface coil (AIR coil; General Electric Signa Architect, General Electric Healthcare, Waukesha, WI, USA) centered at the region of interest. To ensure optimal imaging conditions, fasting was maintained for a minimum of 12 h before the procedure. mpMRI, comprising T1-weighted, T2-weighted, diffusion-weighted, and axial dynamic contrast-enhanced images, was performed in accordance with the guidelines of the European Society of Urogenital Radiology [31]. For prostate imaging using an AIR coil, T2-weighted images were acquired in both the axial and coronal planes, with a section thickness ranging from–3–4 mm and a field of view (FOV) of 22–26 cm. Axial diffusion-weighted single-shot echo-planar imaging was performed with a slice thickness of 4-5 mm and an FOV of 23–25 cm. Dynamic contrast-enhanced imaging was conducted before and after intravenous gadolinium injection (0.1 mmol/kg). The contrast material was administered at an injection rate of 4 mL/s for 5 s. Dynamic examination utilized axial fat-suppressed three-dimensional images with a slice thickness of 3 mm and FOV of 22–26 cm. Patients were classified based on the presence of diffusion restriction and contrast retention in the prostate gland, and prostate volume was measured. The PI-RADS version 2.1 score was determined according to the established protocols [32]. 2D prostate MRE was performed using a multislice, flow-compensated, spin-echo echo-planar imaging (SE-EPI) MRE sequence (General Electric Healthcare, Waukesha, WI, USA). A frequency of 60 Hz is considered low but sufficient for penetration to enable MRE application in solid pelvic organs, as with upper abdominal organs, and is well tolerated by patients. Therefore, a frequency of 60 Hz was selected. Moreover, the current literature recommends 60 Hz for abdominal and pelvic applications [18,33]. Mechanical shear waves at 60 Hz were generated using an active driver system located outside the MRI room. A rigid passive driver secured against the muscle was used to transmit mechanical vibrations from an active transducer into the body. The rigid driver is the standard device used in the FDA-cleared, commercially available implementation of MRE technology (Resoundant Inc., Rochester, MN, USA). Low-frequency longitudinal waves were applied to the prostate using a transducer positioned over the pubic bones before the administration of intravenous contrast. Measurements were performed separately for the central part of the prostate gland, the entire prostate gland, and the lesion. Propagating shear waves were imaged using a motion-sensitized imaging sequence. An SE-EPI sequence was used to acquire axial wave images with the following parameters: repetition time (ms)/echo time (ms), 50/23; continuous sinusoidal vibration, 60 Hz; field of view, 32–42 cm; matrix size, 256 × 64; flip angle, 30°; section thickness, 10 mm; four evenly spaced phase offsets; and four pairs of 60 Hz trapezoidal motion-encoding gradients with zeroth and first moment nulling along the through-plane direction. All processing steps were applied automatically without manual intervention to yield quantitative images of tissue shear stiffness in pascals.

**Figure 2:**
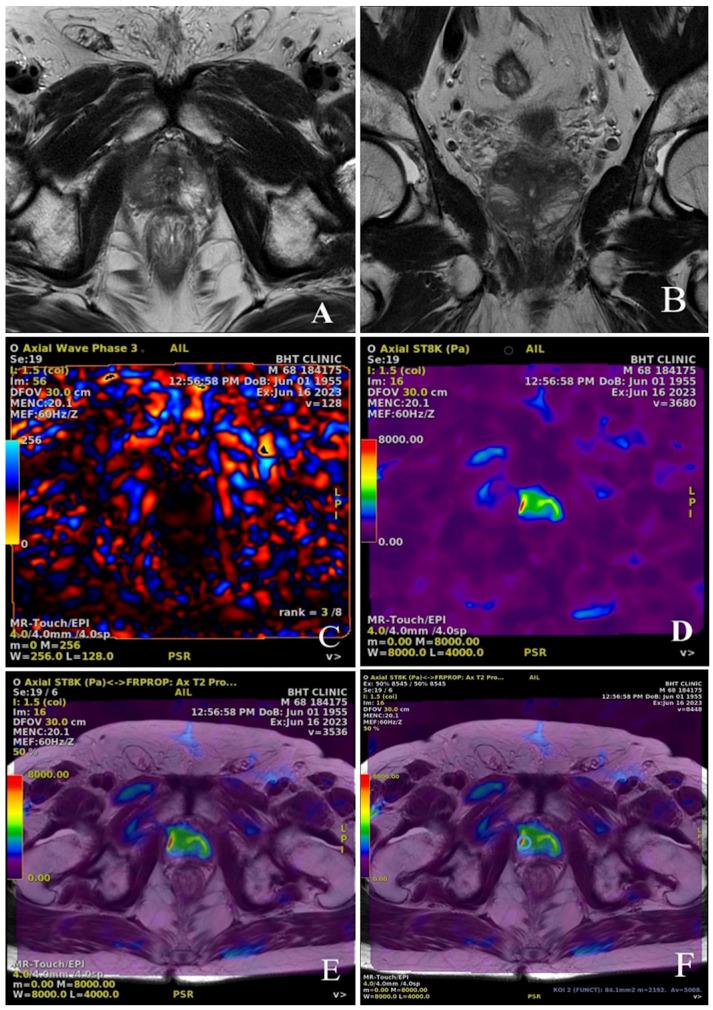
Sample images from a multiparametric prostate MRI examination illustrating the different imaging modalities used for prostate lesion assessment. (**A**) Axial T2-weighted Spin Echo (SE) image and (**B**) Coronal T2-weighted SE image demonstrate a T2 hypointense lesion located at the apex of the prostate gland. (**C**) Phase wave image and (**D**) Elastography wave map of the same lesion, visualizing tissue stiffness distribution. (**E**) Fusion image combining T2-weighted SE and Echo Planar Imaging (EPI)-based elastography, providing anatomical and stiffness information in a single view. (**F**) Stiffness measurement (in Pascals) displayed on the fusion image, where the blue ROI (Region of Interest) circle marks the measured stiffness value of the lesion in the lower right region.

MRE images were processed using commercially available software (Ready View MR-Touch Software version 14.0-6.147, GE Healthcare, Waukesha, WI, US; Resoundant Inc., Rochester, MN, USA) to generate magnitude, phase, and elastograms. The mpMRI and MRE images of the prostate lesions of a 68-year-old participant are presented as examples in Figure 2.

**Figure 3:**
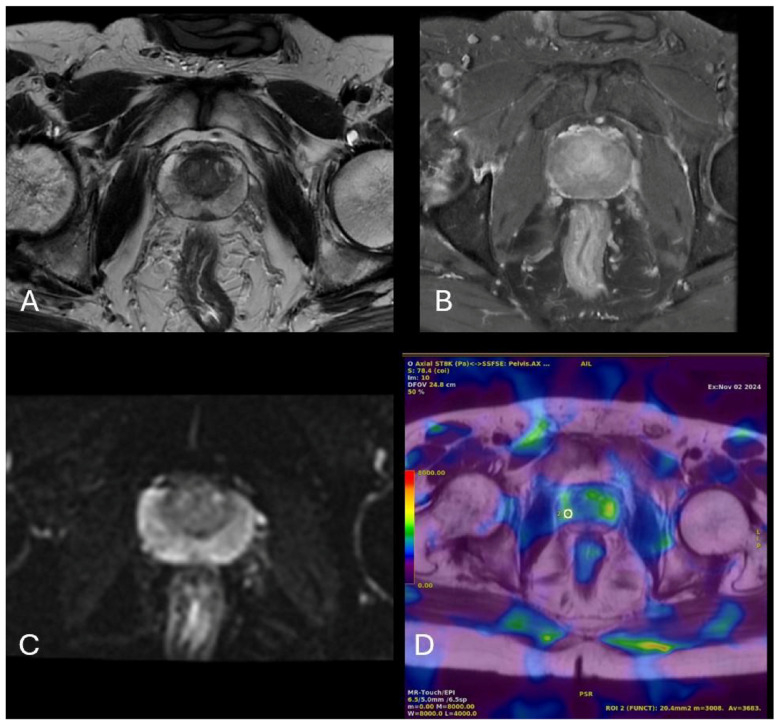
MRI and MRE findings of a 43-year-old patient presenting with dysuria, polyuria, and nocturia and a PSA level of 2.75 ng/mL. Axial T2-weighted imaging shows mild signal loss in the peripheral zone of the prostate (**A**), while no significant contrast enhancement is observed in the prostate parenchyma following intravenous contrast administration (**B**). Additionally, no diffusion restriction was detected on diffusion-weighted imaging (**C**). In the corresponding MRE image (**D**), a region of interest (ROI; white open circle) was placed over a yellow-green coded area representing the stiffest region within the parenchyma, yielding a stiffness value of 3.683 kPa.

MpMRI and MRE images of a 43-year-old patient with a benign prostate lesion are presented in Figure 3.

**Figure 4:**
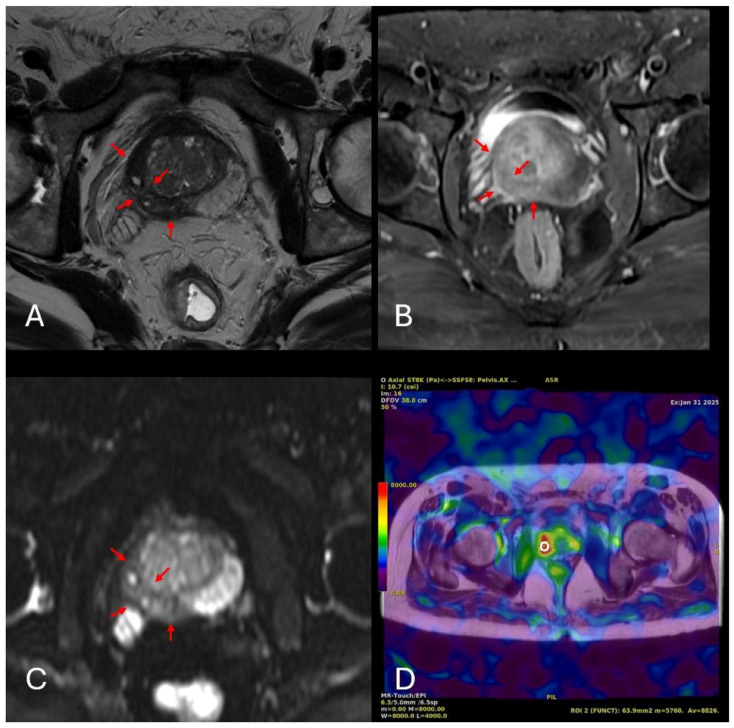
MRI and MRE findings of a 76-year-old patient presenting with dysuria, polyuria, and nocturia, with a PSA level of 5.8 ng/mL. Axial T2-weighted imaging (**A**) demonstrates marked T2 hypointensity in the right peripheral zone (red arrows). Post-contrast imaging (**B**) shows the corresponding contrast enhancement in the same region, whereas diffusion-weighted imaging (**C**) reveals significant diffusion restriction. In the corresponding MRE image (**D**), a region of interest (ROI; white open circle) was placed over a red-coded area representing the stiffest region within the parenchyma, yielding a stiffness value of 8.826 kPa.

MpMRI and MRE images of a 76-year-old patient with a malignant lesion are presented in Figure 4.

To ensure the reproducibility and accuracy of stiffness measurements, all lesions were reviewed by two independent radiologists who were blinded to all clinical, laboratory, and histopathological data. Each radiologist repeated measurements after an interval of at least two weeks to assess intra-observer variability. The final stiffness values were obtained by calculating the mean. Intraclass correlation coefficients (ICCs) were calculated to evaluate both intra-observer and inter-observer consistency, with ICC values ranging between 0.85 and 0.95, indicating excellent reproducibility [34].

### 2.5. Pathological Examination

Prostate biopsies guided by transrectal ultrasound were performed on prostate glands with a PI-RADS (version 2.1) score ≥ 3. Biopsies were performed by experienced surgeons and radiologists at our institution. Analyses were completed within a maximum period of two months after MRE. All specimens were sent to the pathology unit of BHTCLINIC Istanbul Tema Hospital for a thorough pathological examination performed by qualified pathologists according to the International Society of Urological Pathology guidelines [35]. Based on histopathological findings, patients were categorized into three groups: those with benign lesions (benign group), those with premalignant lesions (premalignant group), and those with malignant lesions (malignant group). Patients with prostatitis, scarring, benign prostatic hyperplasia (BPH), and cases in which biopsy was not performed because the PI-RADS score was less than 3 were included in the benign group. Atypical small acinar proliferation (ASAP) and high-grade prostatic intraepithelial neoplasia (HGPIN) are considered premalignant. No other premalignant entities were identified in histopathological findings.

### 2.6. Statistical Analysis

Statistical analyses were performed using SPSS software version 26 (IBM Corp., Armonk, NY, USA). The Shapiro–Wilk test was used to assess the normal distribution of variables. Descriptive statistics included the mean ± standard deviation for normally distributed continuous variables, median (25th–75th percentile) for non-normally distributed continuous variables, and frequencies (percentages) for categorical variables. Between-group analyses of continuous variables were performed using either the ANOVA test (post hoc test: Bonferroni test) or the Kruskall–Wallis H test (post hoc test: Dunn test, depending on the normality of distribution. Between-group analyses of categorical variables were performed using the Chi-square test or Fisher’s exact test. The impact of stiffness metrics derived from MRE on classifying premalignant and malignant lesions was analyzed via logistic regression, using both crude and adjusted models. Adjusted analysis controlled for several covariates, including age, prostate volume, PSA level, and uroflowmetry parameters. The predictive performance of the variables for malignancy was assessed using Receiver Operating Characteristic (ROC) curve analysis. The optimal cutoff points were determined using the Youden index. Sensitivity, specificity, accuracy, positive predictive value, and negative predictive value were calculated based on the optimal cutoff points. Statistical significance was set at *p* < 0.05 (two-tailed) was considered statistically significant.

## 3. Results

The study included 113 men with a mean age of 62.7 ± 7.2 years. Among the 75 patients with benign lesions, 38 had PI-RADS scores below 3, while the remaining 37 with PI-RADS scores ≥ 3 demonstrated pathology findings of scarring in 16 cases, BPH in 11, and prostatitis in 10. Pathology examination revealed premalignant lesions in 15 patients, with ASAP detected in 9 and HGPIN in 6 cases. All malignant lesions were adenocarcinomas. MRE revealed that central gland stiffness averaged 3.4 ± 0.3 kPa and entire-gland stiffness 3.5 ± 0.5 kPa; lesion stiffness demonstrated a median value of 4.0 kPa (IQR: 3.5–7.2 kPa). Conventional mpMRI features were common, with diffusion restriction in 58.4% of cases and contrast retention in 67.3% of cases. The median prostate volume and PSA level were 58 mL (IQR 40–80 mL) and 5.2 ng/mL (IQR 4.0–7.7 ng/mL), respectively. The demographic and clinical characteristics of the patients are presented in Table 1.

**Figure 5:**
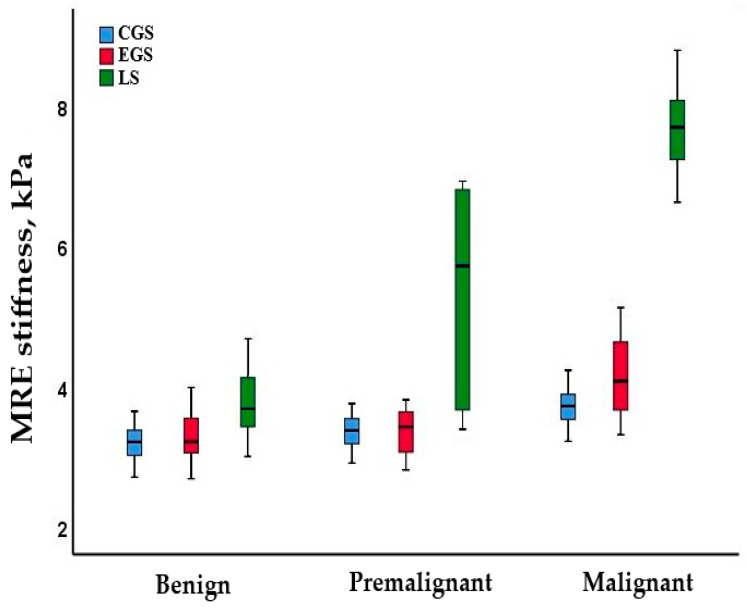
Distribution of central gland stiffness (CGS), entire gland stiffness (EGS), and lesion stiffness (ES) levels measured by MRE in benign, premalignant, and malignant lesions.

The mean age did not differ significantly among the benign, premalignant, and malignant groups. The mean central gland stiffness did not differ between benign and premalignant lesions but was markedly higher in the malignant lesions (benign: 3.3 ± 0.2 vs. premalignant: 3.4 ± 0.2 vs. malignant: 3.6 ± 0.3 kPa; *p* < 0.001). A similar pattern was observed for the entire gland stiffness (benign: 3.3 ± 0.4 vs. premalignant: 3.3 ± 0.4 vs. malignant: 4.1 ± 0.6 kPa; *p* < 0.001). Median lesion stiffness measurements increased stepwise from benign to premalignant to malignant lesions (Benign: 3.6 vs. Premalignant: 5.8 vs. Malignant: 7.7 kPa; *p* < 0.001) (Figure 5).

Diffusion restriction and contrast retention were present in 100% of both premalignant and malignant lesions, whereas they were observed in 37.3% and 50.7% of benign lesions, respectively (both *p* < 0.001). Prostate volume and PSA levels showed no significant differences (*p* = 0.418 and *p* = 0.060, respectively). Uroflowmetry parameters did not differ significantly between the groups (Table 2).

**Figure 6:**
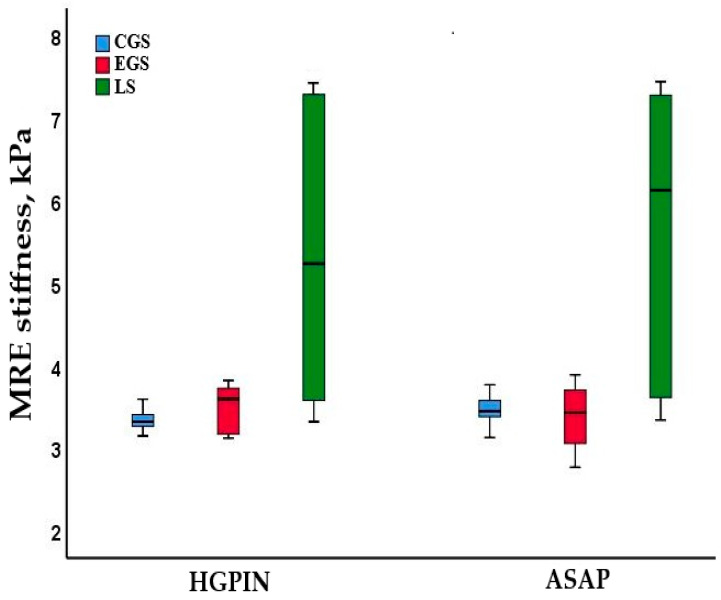
Distribution of central gland stiffness (CGS), entire gland stiffness (EGS), and lesion stiffness (ES) levels measured by MRE in HGPIN and ASAP.

While the median lesion stiffness was higher than other stiffness parameters in all benign subgroups, it did not differ significantly among the subgroups (Figure S1). In patients with premalignant lesions, the mean central gland stiffness did not differ between the HGPIN and ASAP groups (3.3 ± 0.2 vs. 3.4 ± 0.2 kPa, *p* = 0.675). A similar pattern was observed for the entire gland stiffness (3.5 ± 0.3 vs. 3.4 ± 0.4 kPa; *p* = 0.776) and median lesion stiffness (5.2 vs. 6.1 kPa; *p* = 0.481) (Figure 6).

In crude regression analyses, lesion stiffness was the only significant predictor of premalignant lesions versus benign lesions [Odds ratio (OR) = 1.04 per 100 Pa increase; 95% confidence interval (CI), 1.01–1.08; *p* < 0.001], and this association remained after adjusting for age, prostate volume, PSA, and uroflowmetry parameters. When comparing malignant to benign lesions, all three stiffness measures showed strong independent effects: central gland stiffness (adjusted OR = 1.59; 95% CI, 1.21–2.10; *p* = 0.001), entire gland stiffness (adjusted OR = 1.60; 95% CI, 1.25–2.04; *p* < 0.001), and lesion stiffness (adjusted OR = 1.19; 95% CI, 1.09–1.29; *p* < 0.001). Finally, when malignant lesions were compared to premalignant lesions, central gland stiffness (adjusted OR = 1.88; 95% CI 1.14–3.12; *p* = 0.014), entire gland stiffness (adjusted OR = 1.68; 95% CI 1.12–2.52; *p* = 0.013), and lesion stiffness (adjusted OR = 1.20; 95% CI 1.07–1.34; *p* = 0.002) demonstrated significant independent effects (Table 3).

**Figure 7:**
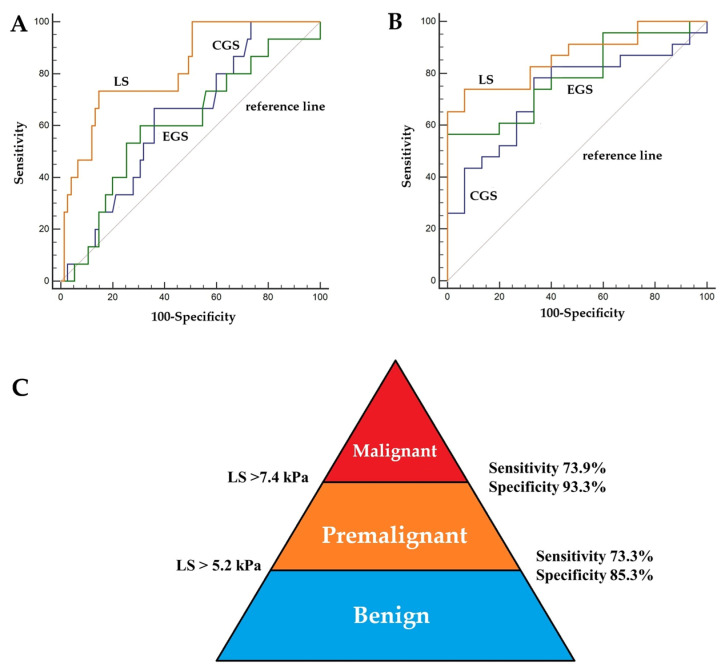
Diagnostic performance of central gland stiffness (CGS), entire gland stiffness (EGS), and lesion stiffness (LS) levels measured by MRE for predicting premalignant (vs. benign) (**A**) and malignant (vs. premalignant) (**B**) lesions. LS exhibited gradually increasing threshold values for distinguish benign, premalignant, and malignant prostate lesions in patients referred for possible prostate cancer (**C**).

In differentiating premalignant lesions from benign lesions, lesion stiffness achieved the highest discriminative performance (AUC 0.82 ± 0.05; accuracy 83.3%), markedly outperforming central gland (AUC 0.63 ± 0.07; accuracy 64.4%) and entire gland stiffness (AUC 0.61 ± 0.08; accuracy 67.8%). Lesion stiffness also demonstrated superior sensitivity (73.3%) and specificity (85.3%) compared with other parameters (Figure 7).

In distinguishing malignant from benign lesions, lesion stiffness demonstrated the highest diagnostic accuracy, with an AUC of 0.95 ± 0.02 and an overall accuracy of 88.8% (sensitivity 91.3%, specificity 88.0%). The entire gland stiffness achieved an AUC of 0.85 ± 0.05 and an accuracy of 79.6% (sensitivity 78.3%, specificity 80.0%), while central gland stiffness was moderately effective (AUC 0.79 ± 0.05; accuracy 74.5%, sensitivity 82.6%. In distinguishing malignant from premalignant lesions, lesion stiffness achieved an AUC of 0.86 ± 0.06 and an accuracy of 82.5% (sensitivity 73.9%, specificity 93.3%), compared with the entire gland stiffness (AUC 0.79 ± 0.07; accuracy 73.6%; sensitivity 56.5%, specificity 100.0%) and central gland stiffness (AUC 0.73 ± 0.08; accuracy 73.7%; sensitivity 78.3%, specificity 66.7%). These results underscore lesion stiffness as the most reliable single MRE metric across all diagnostic distinctions, with consistently higher AUC and accuracy than central or whole gland stiffness (Figure 7) (Table 4).

## 4. Discussion

To the best of our knowledge, this is the first study to comprehensively examine MRE parameters for differentiating benign, premalignant, and malignant lesions in patients with PCa. The central and whole-gland stiffness values were significantly elevated in malignant lesions compared to those in the premalignant and benign lesions, whose stiffness metrics did not differ. Conversely, lesion stiffness increased progressively from benign to premalignant to malignant lesions. Furthermore, elevated lesion stiffness independently predicted premalignant and malignant lesions and displayed stepwise thresholds to distinguish benign, premalignant, and malignant lesions. These findings underscore the clinical potential of lesion-focused MRE quantification as a powerful adjunct to the noninvasive characterization of prostate pathology.

Early diagnosis of PCa is critical for increasing the likelihood of successful treatment and ultimately improving survival [36]. However, there are several challenges in the screening and diagnosis of PCa, primarily due to the limited specificity of conventional or MpMRI diagnostic methods [8,17]. One reason is that diffusion and enhancement characteristics frequently overlap between malignant and non-malignant conditions; benign lesions such as prostatitis or BPH can show restricted diffusion and rapid contrast uptake that mimics carcinoma on MRI [37], and even premalignant lesions (for example, HGPIN or ASAP) may exhibit similarly low apparent diffusion coefficient (ADC) values as true tumors [38]. However, PI-RADS version 2 may still miss clinically significant PCa and underestimate lesion size [39]. Meta-analyses reported a sensitivity of 85–89% and specificity of approximately 71–73% for PI-RADS version 2 [40,41]. Although PI-RADS version 2.1, introduced in 2019, has addressed some of these issues, especially in the transition zone, it still faces challenges, including false-negative findings and inter-observer variability [42,43]. This variability contributes to diagnostic ambiguity, particularly for indeterminate PI-RADS 3 lesions that carry only an ~10–15% likelihood of clinically significant PCa, yet often prompt biopsy due to uncertainty [44]. Consistent with these findings, approximately half of the benign lesions had PI-RADS scores ≥ 3, and most premalignant lesions had PI-RADS scores of 4. Our institutional protocol followed international recommendations, with systematic 12-core biopsy plus 2–4 targeted fusion cores per lesion for all PI-RADS ≥ 3 cases [45]. In our study, the detection rates of clinically significant prostate cancer in PI-RADS 3 and PI-RADS 4 lesions were lower than those reported in large prospective series (12–20% for PI-RADS 3 and 40–60% for PI-RADS 4) [42,44,46]. This discrepancy may be explained by our relatively small sample size, the single-center nature of the study, and referral bias, rather than inadequacy of biopsy technique. In contrast, approximately 35% of malignant lesions were categorized as PI-RADS 4. These diagnostic limitations underscore the urgent need for adjunctive imaging biomarkers that can improve lesion discrimination, particularly in indeterminate or borderline cases.

PCa tissue appears stiffer than healing tissue owing to its higher cell density and increased microvascularization. Destruction of the glandular structure during cancer development leads to tissue hardening during subsequent wound repair [16,47]. Several studies have reported the utility of assessing prostate gland stiffness or intraglandular lesions in the diagnosis of PCa [47,48,49]. Ultrasound (US)-based elastography techniques, particularly shear-wave elastography (SWE), have also been investigated as non-invasive and relatively low-cost tools for prostate cancer detection. SWE has shown promising diagnostic performance, with potential applications in biopsy targeting and risk stratification [48]. However, US elastography is limited by operator dependence, variability in reproducibility, and difficulty in evaluating transition zone nodules due to benign hyperplastic changes [50]. In contrast, MRE provides fully quantitative three-dimensional stiffness maps, demonstrates high inter-observer agreement, and can be integrated into multiparametric MRI workflows [25]. Therefore, while US elastography may represent a more accessible alternative in some healthcare systems, MRE offers superior standardization and whole-gland evaluation, supporting its role as a complementary biomarker in prostate cancer imaging. In this study, we found that MRE provides a quantitative measure of tissue stiffness that can distinguish PCa from benign prostatic tissue. The mean stiffness of PCa lesions was significantly higher than that of benign lesions, confirming that prostate tumors are mechanically stiffer than non-cancerous tissues. This result is consistent with previous MRE studies. Kim et al. reported an average PCa stiffness of ~4.9 kPa versus ~3.6 kPa in normal peripheral zone tissue [25]. Similarly, another recent MRE study using a transpelvic driver found that PCa lesions were substantially stiffer than BPH lesions (approximately 5.9 kPa vs. 4.7 kPa) [19]. These findings align with ex vivo observations that cancerous prostate tissue is approximately two to three times stiffer than normal tissue [50,51], reflecting the same fundamental biomechanical contrast in an in vivo setting. In a study by Deng et al., the stiffness of PCa tissue measured by MRE with a modified driver was significantly higher at all frequencies (except at 60 Hz) than that of non-PCa tissues, including prostatitis and BPH lesions. Their findings demonstrated good diagnostic accuracy for stiffness measurements at higher frequencies, such as 90, 120, and 150 Hz [47]. In another study that examined prostatectomy specimens from individuals with PCa using MRE, a significant increase in the mean stiffness of the tumor tissue compared to that of healthy tissue was detected. Furthermore, MRE can address certain limitations of mpMRI, such as inter-observer variability and low specificity [49]. In our cohort, inter- and intra-observer ICCs ranged from 0.85 to 0.95, confirming excellent repeatability of MRE-derived stiffness. These findings are in line with recent prostate MRE studies reporting excellent inter-reader agreement and support the reliability of quantitative elastography in routine practice [19,28]. Prostate stiffness measured using MRE has also been suggested to aid in noninvasive lymph node metastasis assessment before surgery [52]. Additionally, in an ex vivo study focusing on PCa, MRE exhibited superior performance compared to diffusion-weighted MRI in distinguishing normal tissue from cancerous tissue. The overall conclusion of the authors concluded that diffusion-weighted MRI and MRE provided complementary results [53]. In contrast, Sahebjavaher et al. found that in patients with high-risk scores, the stiffness measured by MRE was significantly different from that of normal tissue [29].

While most studies have reported positive outcomes regarding the predictive efficacy of MRE in PCa, uncertainty remains regarding which specific prostate area measured by MRE holds greater diagnostic value. Several studies have indicated that assessing peripheral zone stiffness using MRE is more valuable for predicting PCa than assessing stiffness in other areas of the prostate [23,24]. Deng et al. suggested that stiffness measurements in the transitional zone via MRE at high frequencies can also serve as a crucial predictor of PCa [47]. It has also been speculated that the outcomes observed in prior studies might be influenced by calcification or blood flow in certain BPH. These factors may mask the stiffness associated with PCa in the transitional zone. Additionally, several previous studies have shown that PCa tends to exhibit higher stiffness than the surrounding normal prostate tissue [50,54].

The studies mentioned above predominantly compared PCa with benign tissue and showed that malignant lesions are significantly stiffer than normal parenchyma or BPH lesions. However, to our knowledge, no published studies have specifically evaluated MRE in HGPIN or ASAP, leaving a gap regarding these premalignant lesions. Biologically, one would anticipate that invasive adenocarcinoma is mechanically stiffer than intraepithelial lesions, as malignant transformation is accompanied by the emergence of a reactive desmoplastic stroma rich in fibroblasts and collagen, which increases tissue rigidity [25]. Premalignant lesions exhibited intermediate stiffness values, with measurements significantly exceeding those of benign lesions but remaining below those associated with malignant lesions; this trend aligns with previous reports in other cancer studies. Notably, despite histological heterogeneity within the benign lesions —including scarring, prostatitis, and BPH—premalignant lesion stiffness consistently remained higher than all benign lesions subgroups, further reinforcing the diagnostic robustness of lesion-level stiffness measurement. However, this benign lesion heterogeneity might explain why central and entire gland stiffness values showed less clear distinction between diagnostic groups. Elastography of breast tissue has shown that “high-risk” precancerous lesions are stiffer than normal benign lesions but softer than carcinoma in situ and invasive cancer [55]. This gradation of stiffness likely reflects progressive microstructural changes during tumorigenesis; as lesions advance toward malignancy, they induce greater cellular density, angiogenesis, and extracellular matrix remodeling (e.g., collagen deposition), all of which increase local tissue rigidity [56,57]. Premalignant lesions may initiate such localized stiffening via mild reactive stromal changes, but these remain confined to the lesion; consequently, the average stiffness of the surrounding glandular tissue (central gland or entire gland) remains similar to that of benign glands. Consistently, in our cohort, only the lesion-specific stiffness metrics showed significant diagnostic power for distinguishing premalignant lesions from benign lesions and for differentiating malignant lesions from premalignant lesions, whereas stiffness measurements of the central gland or whole gland primarily succeeded in separating established cancers [29]. This finding underscores that focal stiffness assessment is more sensitive to early neoplastic changes than global gland stiffness, as small stiff lesions can be masked by the surrounding normal tissue when measuring the organ’s overall elasticity. Thus, lesion-specific stiffness evaluation targeting suspicious areas identified by mpMRI may emerge as a more robust diagnostic parameter compared to general stiffness evaluations of the entire gland or the central region. Therefore, targeted MRE assessments might offer enhanced clinical relevance. Although lesion-level odds ratios were numerically lower than those derived from central or entire gland stiffness, lesion stiffness achieved superior diagnostic accuracy and AUC. This discrepancy can be explained by differences in variance and unit scaling across parameters: gland-level averages, with narrower ranges, yield larger per-unit odds ratios, while lesion-level measurements, with broader distributions, provide greater overall discriminatory power across thresholds. Thus, lesion stiffness, despite lower ORs, emerges as the most clinically meaningful biomarker. From a practical perspective, contemporary prostate cancer diagnostics prioritize the detection of clinically significant tumors (ISUP ≥ 2) while minimizing overdiagnosis of indolent ISUP 1 disease [58]. Although our study was not structured as a screening trial, the observed stepwise stiffness gradient from benign to premalignant to malignant lesions suggests that MRE could add value to mpMRI-based decision-making. This is particularly relevant in diagnostically challenging PI-RADS 3 lesions, where inter-reader variability and limited specificity often complicate biopsy recommendations. Supporting this concept, Chen et al. recently demonstrated that adding MRE-derived stiffness to PI-RADS v2.1 significantly improved the identification of clinically significant prostate cancer [30].

On the other hand, increasing the vibration frequency can shorten the shear wavelength, potentially improving the spatial resolution of stiffness maps and the differentiation of small lesions. Leeet al. implemented a 90 Hz transpelvic driver in prostate MRE, explicitly to obtain shorter-wavelength waves for the smaller prostate organ. They note that higher frequency yields better resolution of tissue structures, helping visualize focal stiffness changes, whereas 60 Hz (the typical liver MRE frequency) was deemed more suitable for a larger organ like the liver [19]. Deng et al. evaluated multi-frequency MRE (60, 90, 120, 150 Hz) in prostate disease and found that image quality and diagnostic performance improved at higher frequencies. Specifically, the confidence of the elastograms (a measure of wave data reliability) was significantly higher at 120–150 Hz than at 60 Hz [47]. Li et al. used tomoelastography (simultaneous 60–80 Hz vibrations) to generate high-resolution stiffness maps of the prostate. Integrating these stiffness metrics with MRI substantially improved cancer detection—raising specificity from 77% with mpMRI alone to 95% when MRE stiffness was included (AUC improvement from 0.85 to 0.95) [24]. A recent study by Kim et al. used 90 Hz with an external acoustic driver, reporting it as an “optimal” frequency for current clinical setups. They observed that 90 Hz waves provided “shorter wavelengths and better resolution, crucial for assessing smaller structures”, aiding in detecting ~1 cm lesions [25]. By contrast, higher frequencies attenuate more rapidly, limiting how much of the prostate gland is reached by sufficient wave amplitude [25]. While higher frequencies (90–150 Hz) may enhance spatial resolution, they are limited by reduced wave penetration and lower patient tolerability in the deep pelvic region. Therefore, 60 Hz was chosen as a balanced and clinically validated frequency addressing both diagnostic performance and patient comfort. Deng et al. reported that MRE at 60 Hz produced reproducible stiffness maps with good diagnostic accuracy and was well tolerated by patients [47]. Similarly, Kim et al. showed that 60 Hz MRE achieved robust diagnostic performance for prostate cancer detection and classification [25]. Moreover, Chen et al. demonstrated that combining 60 Hz MRE stiffness with PI-RADS v2.1 significantly improved the identification of clinically significant prostate cancer, supporting its integration into mpMRI workflows [30]. The choice of 60 Hz in our prostate MRE protocol is well-supported by these trade-offs. It ensures robust wave penetration and high-quality, repeatable stiffness maps in the prostate. Although higher frequencies can sharpen resolution and have shown improved lesion detection in research settings, they come at the cost of greater wave attenuation and technical complexity [19,25].

This study has several noteworthy limitations. First, the single-center design and modest cohort—particularly the small and imbalanced premalignant group—may limit generalizability and reduce statistical power. Second, histologic heterogeneity within the benign category (e.g., scarring, prostatitis, BPH) may have introduced variability in stiffness, especially at the gland level. Third, we did not stratify MRE metrics by lesion location (peripheral vs. transition zone); although zonal microarchitecture may influence stiffness and diagnostic yield, the small and uneven premalignant subgroup precluded a reliable analysis, warranting future studies with standardized zonal mapping and adequate power. Fourth, patients with PI-RADS 2 lesions who did not undergo biopsy were classified as benign. While this carries a theoretical risk of misclassification, it is consistent with guideline-based practice in which PI-RADS 1–2 lesions are not routinely biopsied unless there is strong clinical suspicion [58]. Moreover, several patients in this category declined biopsy despite informed consent, reflecting real-world decision-making and ethical constraints. Fifth, our study relied on biopsy-derived pathology, which inherently carries the risk of sampling error given the multifocal and heterogeneous nature of prostate cancer. Ideally, prostatectomy specimens would provide whole-gland histological correlation with imaging, but these were not available in most patients. However, in routine clinical practice, prostate biopsy remains the internationally accepted gold standard for diagnosis and risk stratification, as highlighted in both the EAU and AUA guidelines [45,58]. In accordance with these standards, our institutional protocol employed a combined approach: all patients with PI-RADS ≥ 3 lesions underwent fusion-targeted biopsy (2–4 targeted cores per lesion) in addition to a 12-core systematic biopsy, an approach designed to maximize detection while minimizing sampling error. Previous imaging studies evaluating novel MRI biomarkers, including MRE, have similarly relied on biopsy as the reference standard [29]. Furthermore, most patients in our cohort were candidates for diagnostic biopsy rather than prostatectomy, and whole-gland specimens were therefore neither ethically nor clinically available. Additionally, the absence of a comparative analysis between 1.5T and 3T MRI scanners and the lack of exploration of the differences between endorectal and external coil use are important limitations that may influence the interpretation of the results. This study did not investigate the impact of varying frequencies on stiffness measurements, an area that remains under discussion in the field of MRE. An other important limitation of our study is that we did not stratify malignant lesions by International Society of Urological Pathology (ISUP) grade group (i.e., ISUP 1 vs. ISUP ≥ 2). This distinction is clinically crucial, as contemporary diagnostic strategies aim to identify clinically significant prostate cancer (ISUP ≥ 2) while minimizing detection of indolent ISUP 1 tumors. Due to the relatively small number of ISUP 1 cases in our cohort, meaningful statistical analysis was not possible. Recent tomoelastography studies supporting the need for further research in this field have demonstrated that quantitative MRE parameters correlate with tumor aggressiveness and may enhance grading discrimination [24]. Collectively, these factors may limit generalizability and transportability; therefore, independent external validation in larger multicenter cohorts—with balanced premalignant sampling, standardized zonal mapping, and sufficient ISUP 1 vs. ISUP ≥ 2 representation—is essential to corroborate our effect sizes and decision thresholds. On the other hand, MRE offers a noninvasive alternative. However, its cost-effectiveness and feasibility for widespread clinical implementation require further investigation. The high costs and technical expertise required for MRE may pose challenges to its routine clinical use, particularly in resource-limited settings. Future studies should address these limitations and assess the practicality of incorporating MRE into standard diagnostic workflows.

## 5. Conclusions

MRE demonstrates considerable potential as a diagnostic tool for prostate cancer evaluation, particularly through lesion-specific stiffness measurements. Our findings indicate that lesion stiffness effectively differentiates benign, premalignant, and malignant prostate lesions, surpassing the diagnostic performance of central and entire gland stiffness metrics. Notably, lesion stiffness exhibited the greatest discriminative power in differentiating premalignant lesions from benign lesions and malignant lesions from premalignant lesions, underscoring its clinical relevance in the early detection and refined risk stratification of prostate cancer. Incorporating MRE-derived lesion stiffness into mpMRI protocols may enhance diagnostic precision and contribute to more personalized approaches to prostate cancer diagnosis, surveillance, and treatment planning.

## Figures and Tables

**Table 1:** Patient characteristics.

Variables	All Population
	*n* = 113
Age, years	62.7 ± 7.2
MRE findings, kPa	
Central gland stiffness	3.4 ± 0.3
Entire gland stiffness	3.5 ± 0.5
Lesion stiffness	3.9 (3.5–7.2)
Diffusion restriction, *n* (%)	66 (58.4)
Contrast retention, *n* (%)	76 (67.3)
Prostate volume, mL	58 (40–80)
PI-RADS, *n* (%)	
PI-RADS 1	18 (15.9)
PI-RADS 2	20 (17.7)
PI-RADS 3	21 (18.6)
PI-RADS 4	39 (34.5)
PI-RADS 5	15 (13.3)
PSA, ng/mL	5.2 (4.0–7.7)
Uroflowmetry findings	
Average flow rate, mL/s	7.2 (4.9–10.9)
Maximum flow rate, mL/s	15.4 (10.8–21.1)
Volume, mL	254.8 (171.7–362.2)

Data are presented as mean ± standard deviation, median (25th–75th percentile), or frequency (percentage). Abbreviations: MRE, magnetic resonance elastography; kPa, kilopascal; PI-RADS, Prostate Imaging-Reporting and Data System; PSA, prostate-specific antigen.

**Table 2:** Distribution of demographic and clinical characteristics of patients with benign, premalignant, and malignant lesions.

Variables	Benign	Premalignant	Malignant	*p*-Value
	*n* = 75	*n* = 15	*n* = 23	
Age, years	62.1 ± 5.9	62.3 ± 9.4	64.8 ± 8.9	0.284
MRE stiffness				
Central gland, kPa	3.3 ± 0.2	3.4 ± 0.2	**3.6 ± 0.3**	<0.001
Entire gland, kPa	3.3 ± 0.4	3.4 ± 0.4	**4.1 ± 0.6**	<0.001
Lesion, kPa	**3.6 (3.4–4.0)**	**5.8 (3.6–7.4)**	**7.7 (7.2–8.1)**	<0.001
Diffusion restriction, *n* (%)	**28 (37.3)**	15 (100.0)	23 (100.0)	<0.001
Contrast retention, *n* (%)	**38 (50.7)**	15 (100.0)	23 (100.0)	<0.001
Prostate volume, mL	56 (39–80)	72 (52–96)	54 (39–79)	0.418
PI-RADS, *n* (%)				
PI-RADS 1	18 (24.0)	0	0	<0.001
PI-RADS 2	20 (26.7)	0	0	
PI-RADS 3	18 (24.0)	3 (20.0)	0	
PI-RADS 4	19 (25.3)	12 (80.0)	8 (34.8)	
PI-RADS 5	0	0	15 (65.2)	
PSA, ng/mL	4.8 (3.6–6.7)	5.2 (4.1–7.9)	6.7 (4.6–20.1)	0.060
Uroflowmetry				
Average flow rate, mL/s	7.2 (4.2–11.1)	6.2 (5.1–8.8)	7.9 (6.1–12.3)	0.479
Maximum flow rate, mL/s	15.4 (9.6–22)	13.3 (11.6–17.4)	17.6 (12.4–21.8)	0.247
Volume, mL	287 (155.2–362.2)	245.5 (225–378.3)	254.8 (186.2–300.1)	0.931

Data are presented as mean ± standard deviation, median (25th–75th percentile), or frequency (percentage). Denominators for all percentages reflect the total number of cases within each pathology group. Values in bold indicate statistically significant differences between groups. Abbreviations: MRE, magnetic resonance elastography; kPa, kilopascal; PI-RADS, Prostate Imaging-Reporting and Data System; PSA, prostate-specific antigen.

**Table 3:** Crude and adjusted regression analyses of MRE stiffness parameters for the prediction of premalignant and malignant prostate lesions.

Variables	Crude Regression		Adjusted Regression	
	OR (95% CI)	*p*-Value	OR (95% CI)	*p*-Value
Premalignant vs. benign				
Central gland stiffness	1.21 (0.94–1.55)	0.133	1.16 (0.87–1.55)	0.306
Entire gland stiffness	1.10 (0.95–1.29)	0.214	1.11 (0.93–1.32)	0.246
Lesion stiffness	1.04 (1.01–1.08)	<0.001	1.04 (1.01–1.09)	0.022
Malignant vs. benign				
Central gland stiffness	1.61 (1.27–2.04)	<0.001	1.59 (1.21–2.10)	0.001
Entire gland stiffness	1.38 (1.20–1.59)	<0.001	1.60 (1.25–2.04)	<0.001
Lesion stiffness	1.15 (1.08–1.24)	<0.001	1.19 (1.09–1.29)	<0.001
Malignant vs. Premalignant				
Central gland stiffness	1.44 (1.03–2.00)	0.002	1.88 (1.14–3.12)	0.014
Entire gland stiffness	1.27 (1.07–1.51)	<0.001	1.68 (1.12–2.52)	0.013
Lesion stiffness	1.10 (1.03–1.18)	<0.001	1.20 (1.07–1.34)	0.002

The crude models included each MRE stiffness parameter alone. The adjusted models were additionally controlled for age, prostate volume, PSA level, and uroflowmetry parameters. Abbreviations: CI, confidence interval; OR, odds ratio.

**Table 4:** Diagnostic performance of MRE parameters in differentiating premalignant and malignant prostate lesions.

ROC Results	MRE Stiffness			mpMRI	Combination *
	Central Gland	Entire Gland	Lesion	PI-RADS	
Premalignant vs. Benign					
AUC ± SE	0.63 ± 0.07	0.61 ± 0.08	0.82 ± 0.05	0.82 ± 0.05	0.85 ± 0.05
95% CI	0.52–0.73	0.49–0.71	0.73–0.90	0.73–0.89	0.75–0.94
Sensitivity, %	66.7	60.0	73.3	80.0	93.3
Specificity, %	64.0	69.3	85.3	74.7	69.3
Accuracy, %	64.4	67.8	83.3	75.5	73.3
PPV, %	27.0	28.1	50.0	38.7	37.8
NPV, %	90.6	89.7	94.1	94.9	98.1
Cut-off value	>3.3 kPa	>3.4 kPa	>5.2 kPa	>3	PI-RADS > 3 & LS > 5.2 kPa
*p*-value	0.064	0.293	<0.001	<0.001	<0.001
Malignant vs. Benign					
AUC ± SE	0.79 ± 0.05	0.85 ± 0.05	0.95 ± 0.02	0.96 ± 0.02	0.92 ± 0.04
95% CI	0.70–0.87	0.76–0.91	0.89–0.99	0.89–0.99	0.84–0.99
Sensitivity, %	82.6	78.3	91.3	100.0	91.3
Specificity, %	72.0	80.0	88.0	74.7	92.0
Accuracy, %	74.5	79.6	88.8	80.6	91.8
PPV, %	47.5	54.5	70.0	54.8	77.8
NPV, %	93.1	92.3	97.1	100.0	97.2
Cut-off value	>3.4 kPa	>3.7 kPa	>6.9 kPa	>3	PI-RADS > 3 & LS > 6.9 kPa
*p*-value	<0.001	<0.001	<0.001	<0.001	<0.001
Malignant vs. Premalignant					
AUC ± SE	0.73 ± 0.08	0.79 ± 0.07	0.86 ± 0.06	0.86 ± 0.06	0.87 ± 0.04
95% CI	0.56–0.86	0.62–0.90	0.71–0.95	0.71–0.95	0.73–0.96
Sensitivity, %	78.3	56.5	73.9	65.2	91.3
Specificity, %	66.7	100.0	93.3	100.0	93.3
Accuracy, %	73.7	73.6	82.5	78.9	92.1
PPV, %	78.3	100.0	94.4	100.0	95.5
NPV, %	66.7	60.0	70.0	65.2	87.5
Cut-off value	>3.5 kPa	>3.9 kPa	>7.4 kPa	>4	PI-RADS > 4 & LS > 7.4 kPa
*p*-value	0.006	<0.001	<0.001	<0.001	<0.001

* represents the combination of PI-RADS and lesion stiffness parameters. Abbreviations: AUC, Area under ROC curve; CI, Confidence interval; MRE, Magnetic resonance elastography; NPV, Negative predictive value; PI-RADS, Prostate Imaging-Reporting and Data System; PPV, Positive predictive value.

## Data Availability

The data that support the findings of this study are available on request from the corresponding author. The data are not publicly available due to legal or ethical reasons.

## Supplementary Materials

The following supporting information can be downloaded at: https://www.mdpi.com/article/10.3390/diagnostics15202603/s1, Figure S1: Distribution of MRE-derived stiffness values in benign group.

## Abbreviations

The following abbreviations are used in this manuscript:

MRE	Magnetic resonance elastography
PSA	Prostate-specific antigen
PI-RADS	Prostate Imaging Reporting and Data System
PCa	Prostate cancer
BPH	Benign prostatic hyperplasia
mpMRI	Multiparametric magnetic resonance imaging
HGPIN	High-grade prostatic intraepithelial lesions
FOV	Field of view
SE-EPI	Spin-echo echo-planar imaging
ASAP	Atypical small acinar proliferation
ROC	Receiver Operating Characteristic
OR	Odds ratio
CI	Confidence interval
AUC	Area under ROC curve
NPV	Negative predictive value
PPV	Positive predictive value
ADC	Apparent diffusion coefficient
