# Supplementary figures and images for: Lesion Stiffness Measured by Magnetic Resonance Elastography: A Novel Biomarker for Differentiating Benign, Premalignant and Malignant Prostate Lesions
Source: Diagnostics (Basel). 2025 Oct 16;15(20):2603. doi: 10.3390/diagnostics15202603 (PMC12563254; doi:10.3390/diagnostics15202603)

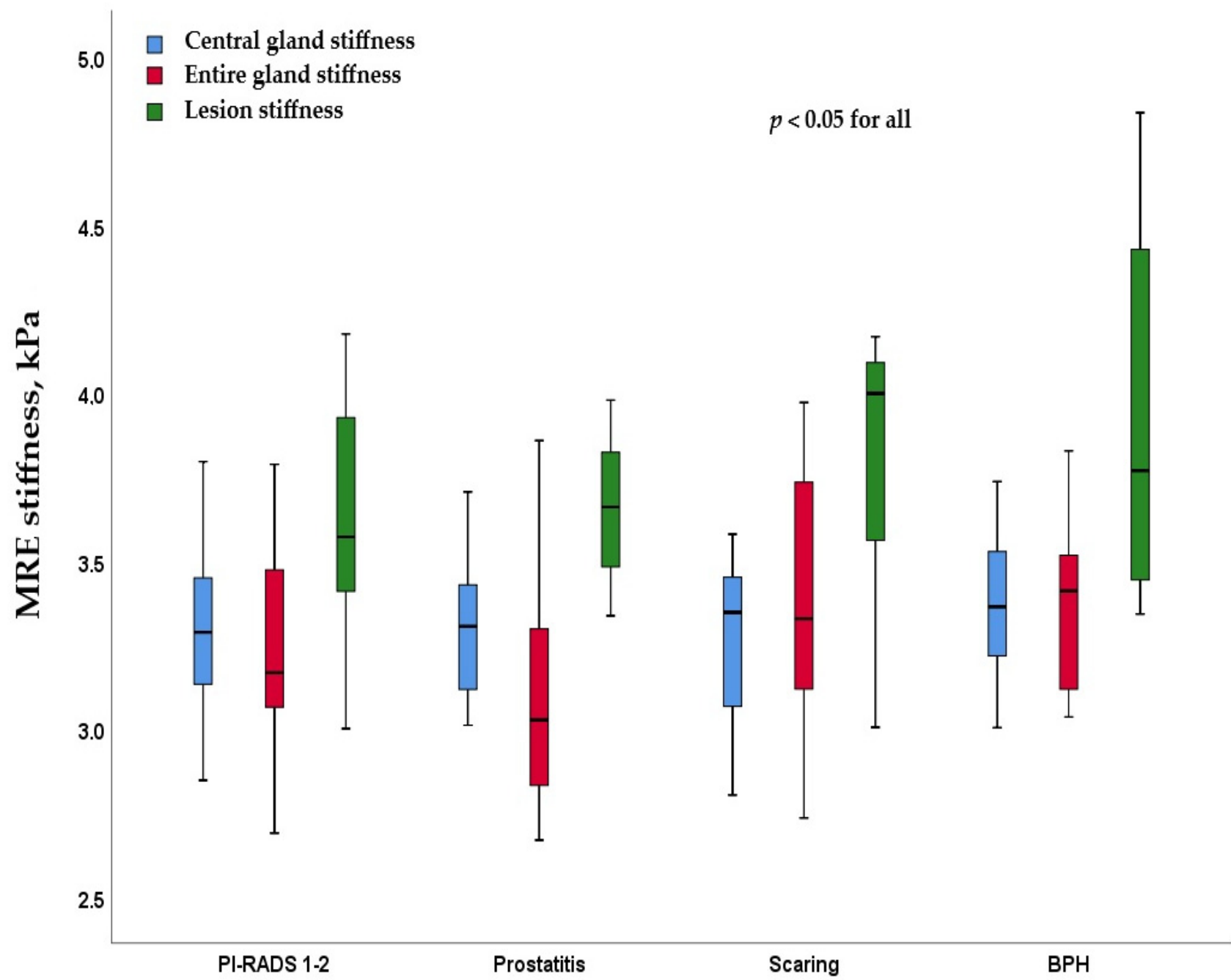

Supplement Figure S1. Distribution of MRE-derived stiffness values in benign group.

Supplement: Supplementary file 1 [file diagnostics-15-02603-s001.zip › diagnostics-3797973-supplementary.pdf]
